# Investigation of Tissue-Specific Expression and Functions of MLF1-IP during Development and in the Immune System

**DOI:** 10.1371/journal.pone.0063783

**Published:** 2013-05-28

**Authors:** Xuehai Wang, Martin Marcinkiewicz, Yaned Gatain, Maxime Bouchard, Jianning Mao, Michel Tremblay, Noriko Uetani, Silva Hanissian, Shijie Qi, Jiangping Wu, Hongyu Luo

**Affiliations:** 1 Laboratoire d’Immunologie, Centre de Recherche, Centre hospitalier de l’Université de Montréal (CRCHUM) – Hôpital Notre-Dame, Montreal, Quebec, Canada; 2 Service de Nephrologie, Centre de Recherche, Centre hospitalier de l’Université de Montréal (CRCHUM) – Hôpital Notre-Dame, Montreal, Quebec, Canada; 3 Cytochem Inc., Montreal, Quebec, Canada; 4 Rosalind and Morris Goodman Cancer Research Centre (GCRC), McGill University, Montreal, Quebec, Canada; 5 Department of Neurosurgery, University of Tennessee, Memphis, Tennessee, United States of America; University of California, Riverside, United States of America

## Abstract

Myeloid leukemia factor 1-interacting protein (MLF1-IP) has been found to exert functions in mitosis, although studies have been conducted only in cell lines up to now. To understand its roles during ontogeny and immunity, we analyzed its mRNA expression pattern by *in situ* hybridization and generated MLF1-IP gene knockout (KO) mice. MLF1-IP was expressed at elevated levels in most rudimentary tissues during the mid-gestation stage, between embryonic day 9.5 (e9.5) and e15.5. It declined afterwards in these tissues, but was very high in the testes and ovaries in adulthood. At post-natal day 10 (p10), the retina and cerebellum still expressed moderate MLF1-IP levels, although these tissues do not contain fast-proliferating cells at this stage. MLF1-IP expression in lymphoid organs, such as the thymus, lymph nodes, spleen and bone marrow, was high between e15.5 and p10, and decreased in adulthood. MLF1-IP KO embryos failed to develop beyond e6.5. On the other hand, MLF1-IP^+/−^ mice were alive and fertile, with no obvious anomalies. Lymphoid organ size, weight, cellularity and cell sub-populations in MLF1-IP^+/−^ mice were in the normal range. The functions of MLF1-IP^+/−^ T cells and naïve CD4 cells, in terms of TCR-stimulated proliferation and Th1, Th17 and Treg cell differentiation *in vitro,* were comparable to those of wild type T cells. Our study demonstrates that MLF1-IP performs unique functions during mouse embryonic development, particularly around e6.5, when there was degeneration of epiblasts. However, the cells could proliferate dozens of rounds without MLF1-IP. MLF1-IP expression at about 50% of its normal level is sufficient to sustain mice life and the development of their immune system without apparent abnormalities. Our results also raise an intriguing question that MLF1-IP might have additional functions unrelated to cell proliferation.

## Introduction

Myeloid leukemia factor 1-interacting protein (MLF1-IP) has several other designations: Kaposi’s sarcoma-associated herpes virus latent nuclear antigen-interacting protein 1 (KLIP1); centromere protein of 50 kDa (CENP50); centromere protein U (CENPU); interphase centromere complex protein 24; polo-box-interacting protein 1 (PBIP1).

Although MLF1-IP cDNA was identified by the human genome project, its initial functional attributes were reported by 2 groups. Yeast 2-hybrid screening by Pan et al. discerned that the protein interacts with the latent nuclear antigen of Kaposi’s sarcoma-associated herpes virus [1], while Hanissian et al. reported that it interacts with myeloid leukemia factor 1 [2]. More earnest functional characterization ensued in subsequent years. Minoshima et al. discovered, again by yeast 2-hybrid screening, that MLF1-IP is a constitutive component of centromeres and named it CENP50 [3]. It interacts with a Rho GTPase-activating protein, MgcRacGAP. MLF1-IP-deficient cells are viable but undergo delayed mitosis and severe mitotic defects, such as chromosome misalignment and premature sister chromatid separation, demonstrating, for the first time, that it is located in centromeres and plays a role in mitosis. Employing multiple tandem affinity purification, Foltz et al. identified MLF1-IP in CENP-A nucleosome-associated complexes in centromeres [4]. Yeast 2-hybrid screening by Kang et al. determined that MLF1-IP is associated with polo-like kinase 1 (Plk1), and its T78 is a substrate of this kinase [5]. MLF1-IP T78 phosphorylation creates self-tethering sites for interaction with Plk1, and such interaction is crucial for Plk1 recruitment to interphase and mitotic kinetochores. Lack of MLF1-IP T78 phosphorylation induces a chromosome congression defect and compromises the spindle checkpoint. Later in mitosis, Plk1 also evokes MLF1-IP degradation in a T78 phosphorylation-dependent manner. Recently, the same group established that MLF1-IP interacts directly with CENP-Q, and these interactions are necessary for the centromere localization of both CENP-Q and MLF1-IP to form ternary complexes with Plk1 [6]. Hua et al. determined that MLF1-IP also interacts with Hec1, a kinetochore core component [7]. It not only binds directly to microtubules but also shows cooperative microtubule binding with Hec1. MLF1-IP knockdown results in impaired kinetochore-microtubule attachment.

All the above-described MLF1-IP functions are based on *in vitro* experiments in cell lines. It is not known whether MLF1-IP has functions other than that in mitosis. Also, no *in vivo* study has explored whether MLF1-IP is essential in mouse development. In our investigation, we conducted *in situ* hybridization (ISH) analysis to assess the tissue-specific expression of MLF1-IP during ontogeny with a view to identifying its functions. We also generated MLF1-IP gene null mutation in mice to evaluate the importance of this gene *in vivo* and *in vitro*.

## Materials and Methods

### ISH

Full-length 1.25-kb MLF1-IP cDNA in pENBR/SD/D-TOPO was isolated with NotI/SalI and sub-cloned into NotI/SalI sites of pSPORT6 [2]. The resulting plasmid was named pSPORT6-MLF1-IP and was employed as a template for sense and anti-sense riboprobe synthesis, using SP6 and T7 RNA polymerase for both ^35^S-UTP and ^35^S-CTP incorporation [8].

Tissues were frozen in −35°C isopentane and kept at −80°C until sectioned. We studied 10-µm thick cryostat-cut slices by ISH and x-ray film autoradiography, as outlined previously [8].

In situ hybridization in embryos was performed as described using digoxigenin-labeled RNA probes [9]. Hybridization was carried out with probes for *Lim1* and *Otx2* [10], [11].

Hematoxylin and eosin staining was performed using standard procedures.

### Generation of MLF1-IP Gene Knockout (KO) Mice

**Figure 1:**
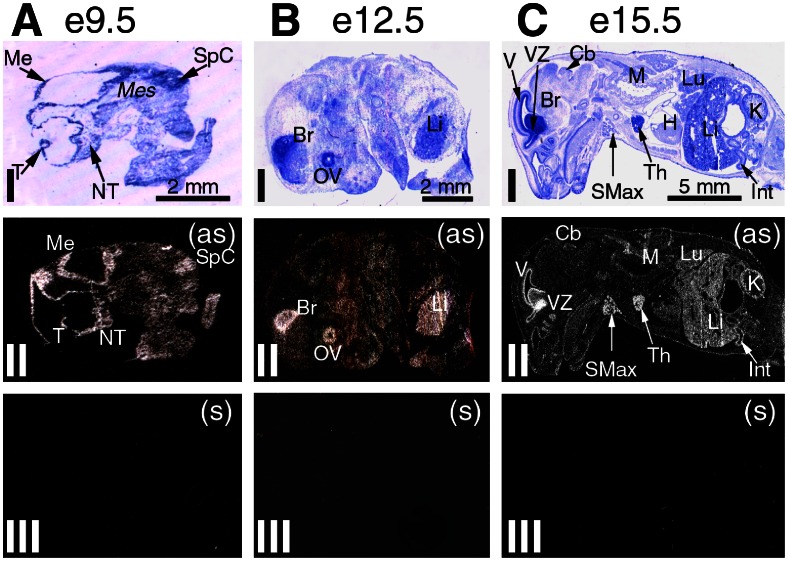
ISH analysis of MLF1-IP expression in mid-gestation embryos e9.5, e12.5 and e15.5. Sagittal sections of the embryos are shown as follows: (I) crystal violet staining under bright-field illumination, and (II) antisense and (III) sense (control) hybridization x-ray film under dark-field illumination. Column A: an e9.5 embryo with hybridization signal seen over a rudimental brain and spinal cord and with less signal within mesenchymal centers. Column B: an e12.5 embryo with hot spots of MLF1-IP hybridization in the brain, optic vesicle and liver. Column C: an e15.5 embryo showing further differentiation of MLF1-IP expression distribution. Strong hybridization signal is observed in the brain ventricular zone rich in neuroblasts and ventricle wall, submaxillary gland and thymus. Lung parenchyma, liver, kidney marginal zone and growing intestine showed moderate-level MLF1-IP mRNA labelling. Original magnifications: (A) 6.5X; (B) 4.5X and (C) 2.6X. Abbreviations used in Figures 2, 3, 4: Adr – adrenal gland; Al – alveolar bone; AR – aortic root; BM – bone marrow; Br – brain; C – calvaria bone; Cb – cerebellum; CL – corpus luteum; Cx – cortex; DA – dorsal aorta; F – follicles; Fe – femur, bone and bone marrow; H – heart; Int – intestine; K – kidney; LF – lymph follicle; Li – liver; LInt – Large intestine; LN – lymph node; Lu – lung; M – muscles, striated; Me – mesencephalon; Med – medulla; *Mes* – mesenchyme; NT – nasal tip; Ov – ovary; OV – optic vesicle; R – retina; Ri – ribs; SfT – seminiferous tubules; Sk – skin; SMax – submaxillary gland; SpC – spinal cord; St – stomach; T – telencephalon; Tes – testes; Th – thymus; V – ventricular wall; Ve – vertebrae; VZ – ventricular zone; WP – white pulp; (as) – antisense; (s) – sense.

A polymerase chain reaction (PCR) fragment, amplified on the MLF1-IP cDNA sequence in pSPORT6-MLF1-IP, served as probe to isolate genomic BAC DNA clone 325N12 from the 129/sv mouse BAC genomic library RPCI-22. The targeting vector was constructed by recombination and routine cloning methods, with a 10.6-kb MLF1-IP genomic fragment from clone 325N12, as illustrated in Fig. 1A [12]. A 2.39-kb BamHI/HpaI genomic fragment containing exon 1 was replaced by a 1.1-kb Neo cassette from pMC1Neo Poly A. The final targeting fragment was excised from its cloning vector backbone by NotI digestion and electroporated into R1 embryonic stem (ES) cells for G418 selection [13].

Southern blotting with a probe corresponding to the 5′ sequence outside the targeting region, as illustrated in Figure 1A (red square), screened for and confirmed gene-targeting in ES cells and eventually in mouse tail DNA. The targeted allele showed a 6.8-kb NcoI band, and the wild type (WT) allele, a 10-kb NcoI band (Fig. 1A). PCR was adopted for routine genotyping of the targeted allele(s). The following PCR conditions were applied: 4 min at 95°C, followed by 35 cycles of 30 s at 94°C, 30 s at 58°C, and 30 s at 72°C, with final incubation at 72°C for 10 min. KO forward primer 5′-GCC AAG TCC AAC GTC TTG AT-3′ and reverse primer 5′-CTC TTG CAA AAC CAC ACT GC-3′ detected a 202-bp fragment from the targeted allele. WT forward primer 5′-TTA CTG CGG TAT TCT GTG CTG GGA-3′ and reverse primer 5′ ATC TAC TTG CAT CTG CCT CCG AGT-3′ detected a 588-bp fragment from the WT allele.

The targeted ES cell clones were injected into C57BL/6 blastocysts. Chimeric male mice were mated with C57BL/6 females to establish mutated MLF1-IP allele germline transmission. All mice were housed under specific pathogen-free conditions and the project including the generation of the KO mice was approved by the Institutional Animal Protection Committee of the CRCHUM (NO9055JWs).

### Real Time Reverse Transcription-quantitative PCR (RT-qPCR)

MLF1-IP mRNA in cells or tissues from KO, heterozygous (HET) and WT mice was measured by RT-qPCR. Total RNA was extracted with TRIzol® (Invitrogen, Carlsbad, CA, USA) and then reverse-transcribed with Superscript II™ reverse-transcriptase (Invitrogen). The forward and reverse primers were 5′-GCA AGG AGA AGT TTG AGA TAC TCG GG-3′ and 5′-CCA GCT TTC TGT TTC CTG GAA TAT GTG C-3′, respectively. A 91-bp product was detected with the following amplification program: 95°C×15 min, 1 cycle; 94°C×15 s, 55°C×30 s, 72°C×30 s, 35 cycles.

Oct-4 and Nanog mRNA in ES cells was quantified by RT-qPCR. For Oct-4, a forward primier 5′- CCT ACA GCA GAT CAC TCA CAT C -3′ and a reverse primer 5′- GCC GGT TAC AGA ACC ATA CTC -3′ were employed; for Nanog, a forward primier 5′- TGC AAG AAC TCT CCT CCA TTC -3′ and a reverse primer 5′- CGC TTG CAC TTC ATC CTT TG -3′ were used.

β-actin mRNA levels were measured as internal controls; the forward and reverse primers were 5′-TGG TAC CAC AGG CAT TGT GAT-3′ and 5′-TGA TGT CAC GCA CGA TTT CCC T-3′, respectively, with the same amplification program as for MLF1-IP mRNA.

RT-qPCR was performed in triplicate, and the results were expressed as the signal ratios of MLF1-IP/β-actin.

RT-qPCR was also undertaken for embryo genotyping. Embryos were digested at 55°C for 4 h in 2 µl digestion buffer (proteinase K, 0.01% gelatin, 0.005% NP-40, 20 mM Tris (pH 8.35), 40 mM KCl, 0.5 mM MgCl_2_). Forward primer 5′-GCC TGG AAT GTT TCC ACC CAA TGT-3′ and reverse primer 5′-CTG CGT GTT CGA ATT CGC CAA TGA-3′ detected a 144-bp fragment from the KO allele, while forward primer 5′-TTA CTG CGG TAT TCT GTG CTG GGA-3′ and reverse primer 5′ CTT CCA AGG CGC ACC TTT CCA AAT-3′ detected a 193-bp fragment from the WT allele. PCR conditions were as follows: 50°C×2 min, 1 cycle; 95°C×2 min, 1 cycle; 94°C×10 s, 58°C×20 s, 72°C×20 s, 35 cycles.

### ES Cell Culture and Small Interfering RNA (siRNA) Transfection

ES cells were maintained in DMEM supplemented with 15% fetal bovine serum, 1×MEM non-essential amino acids, 2mM L-glutamine (WISENT, St. Bruno, Quebec, Canada), 100 µM 2-mercaptoethanol (Sigma, St. Louis, MO, USA) and 1000 U/ml leukemia inhibitory factor (Chemicon, Billerica, MA, USA). siRNAs to MLF1-IP and negative control siRNAs were synthesized by Integrated DNA Technologies (Coralville, IA, USA). The sequences of the MLF1-IP-specific siRNA and control siRNA are listed in Table 1. ES cells were transfected with a mix of 2 pairs of siRNAs (each pair at a final concentration of 30 nM), with FuGENE HD X-tremeGENE siRNA Transfection Reagent (Roche Applied Science, Mannheim, Germany). The transfected ES cells were further cultured for 48 h. The MLF1-IP mRNA knockdown and mRNA expression of Oct-4 and Nanog were measured by RT-qPCR.

**Table 1:** Sequences of siRNA specific to MLF1-IP and control siRNA.

		Sets of siRNA sequences	
Gene		Sense sequences	Antisense sequences
**Control**		5′-rCrGrU rUrArA rUrCrG rCrGrU rArUrA rArUrA rCrGrCrGrUA T-3′	5′-rArUrA rCrGrC rGrUrA rUrUrA rUrArC rGrCrG rArUrU rArArC rGrArC-3′
**MLF1-IP**	Duplex 1:	5′-rGrGrA rArUrA rArArG rArUrU rArGrU rCrArG rArArUrArUrG T-3′	5′-rArCrA rUrArU rUrCrU rGrArC rUrArA rUrCrU rUrUrA rUrUrC rCrUrC-3′
	Duplex 2:	5′-rArGrA rGrUrA rGrArA rUrCrU rGrArA rArGrU rUrGrUrAAT C-3′	5′-rGrArU rUrArC rArArC rUrUrU rCrArG rArUrU rCrUrA rCrUrC rUrUrU-3′

### Flow Cytometry

Single cell suspensions from the thymus, lymph node and spleen were prepared and stained for flow cytometry [14].

### T-cell Proliferation

T-cell proliferation was assessed by ^3^H-thymidine uptake [15].

### Naïve T-cell Differentiation into Th1, Th17 and Treg Cells

The method has been described in a previous publication [16].

## Results

### MLF1-IP Expression during Ontogeny According to ISH

**Figure 2:**
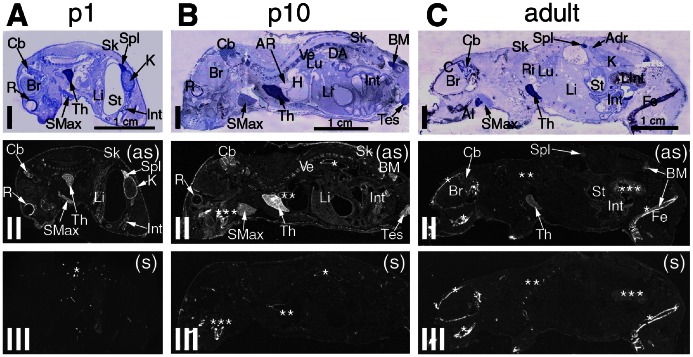
ISH analysis of MLF1-IP mRNAS expression in p1, p10 and adult mice. Sagittal sections of p1, p10 and adult mice were stained with crystal violet (I), antisense probes (II) and sense probes (control) (III) as described in Figure 1. Column A: sections of a newborn (p1) mouse with MLF1-IP mRNA labelling seen in the retina, cerebellum, submaxillary gland, thymus, liver, intestine and kidney marginal zone. Column B: sections of a p10 mouse displaying various levels of MLF1-IP expression in different organs, including bone marrow within vertebrae and testis. Non-specific hybridization is noted in dorsal aorta (*), aortic root (**) and alveolar bone (***) according to sense probe hybridization. Column C: sections of an adult mouse showing low-level MLF1-IP mRNA in the cerebellum, thymus, spleen and bone marrow. There is no detectable hybridization in the liver and kidney. Non-specific hybridization is seen in the femoral, skull alveolar bones (*), ribs (**) and in the content of the large intestine (***). Original magnifications: (A) 1.5X; (B) 1.3X and (C) 1.0X.

**Figure 3:**
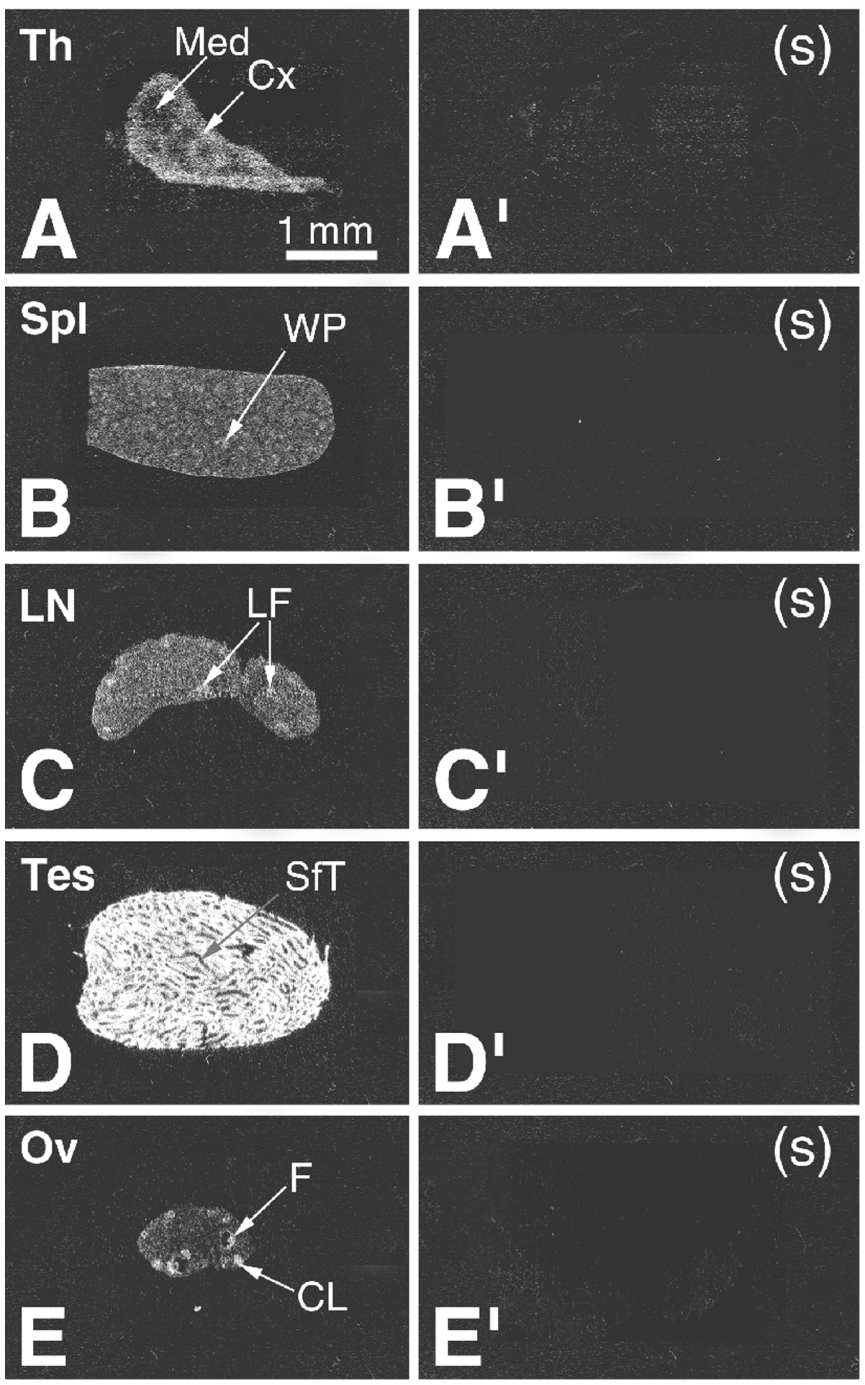
ISH analysis of MLF1-IP expression in individual adult tissues. X-ray autoradiography was under dark-field illumination. The left column shows antisense hybridization, and the right column, sense (control) hybridisation. A and A′: thymus, showing ISH signal mostly in the cortex and less in the medulla. B and B′: spleen, very low-level ISH signal in the white pulp. C and C′: Lymph node, low-level ISH labelling in the lymphatic follicles; D and D′: testis, high-level MLF1-IP expression in seminiferous tubules. E and E′: MLF1-IP mRNA detectable in the follicles and corpus luteum. Original magnification: 10X.

So far, detailed tissue-specific expression of MLF1-IP during ontogeny has not been investigated. Our study provides ISH evidence of spatially- and temporally-restricted MLF1-IP expression patterns in mice from mid-gestation through adulthood. Based on exposure time necessary for autoradiograms (optimal time: 10 days for x-ray films), MLF1-IP mRNA belonged to a class of very low abundant mouse mRNAs. A summary appears in Table 2, and details are given in Figures 1, 2, 3. Generally speaking, rudiments of multiple tissues (the brain, liver, optic vesicle and thymus) displayed relatively high MLF-IP mRNA levels, which peaked in the mid-gestation stage. At mid-gestation, on embryonic day 9.5 (e9.5), rudiments of multiple tissues, except for the mesenchymal region, displayed relatively high MLF1-IP mRNA levels (Fig. 1A-II); expression was particularly high in the central nervous system (CNS) structures, such as the mesencephalon, telencephalon, nasal tip and spinal cord (Fig. 1A-II). On day e12.5, MLF1-IP-positive areas in the CNS were limited to a small region in the brain and optic vesicle (Fig. 1B-II). By day e15.5, ventricular wall, ventricular zone and cerebellum displayed MLF1-IP mRNA labelling (Fig. 1C-II). A number of tissues displayed changing levels of MLF1-IP expression through several stages. In the liver, a moderate level of MLF1-IP expression was evident on day e12.5, and it progressively diminished through e15.5, post-natal day 1 (p1), p10 to decline at adulthood (Figs. 1 and 2), showing a pattern reminiscent to that of erythropoiesis genes: such genes have peak expression at prenatal stages, before their function is fully taken by a bone marrow cells. In this respect, MLF1-IP was observed in postnatal p10 and adult mice bone marrow within vertebrae and long bone cavities (Fig. 2B and C), suggesting its possible involvement in the erythropoiesis. Transient expression in the kidney nephrogenic zone was observed from e15.5 to p1. There was no evident MLF1-IP expression in the adult kidney (Fig. 2C-I and II). Long-lasting expression is observed in the intestine, from e15.5 until adulthood. The skin manifested peak MLF1-IP expression on p10. From p1 until adulthood, MLF1-IP was expressed in the retina and cerebellum (Figs. 2A-II, B-II and C-II). MLF1-IP expression peaked in the submaxillary gland between e15.5 and p1 (Figs. 2A-II and 2B-II), but its expression in this gland was not evident in adults (Fig. 2C-II). In the immune system, the thymus and spleen presented moderate to high MLF1-IP expression from e15.5 to p10; it declined to a detectable level in adulthood (Figs. 1 and 2), and was mainly concentrated in the thymic cortex (Fig. 3A), spleen white pulp (Fig. 3B) and lymph node follicles (Fig. 3C). In the reproductive system, MLF1-IP expression was already moderately high in the testes on p10; it became relatively high in adult testes (Fig. 3D). Moderate to high MLF1-IP expression was found in growing follicles and corpus luteum of the ovaries (Figs. 3D and 3E).

**Table 2:** Summary of MLF1-IP mRNA expression in various tissues and organs during mouse ontogeny according to ISH.

	Embryonic stage					
Tissue	e9.5	e12.5	e15.5	p1	p10	Adult
Brain and spinal cord	+++	++++	−/+++	−/+	−/+	−/+
Cerebellum	−	−	−	+	++	±
Eyes	+	++	ne	+++	++	ne
Olfactory neuroepithelium			+	ne	ne	ne
Liver	+	+++	++	+	+	±
Kidneys	−	+	++	+++	ne	ne
Submaxillary gland	−	−	++	++	+	±
Vertebrae	−	−	−	+	++	−
Calvaria	−	−	−	+	++	−
Thymus	−	−	+++	++	++	±
Spleen			ne	+++	ne	±
Lymph nodes					ne	±
Ovaries					ne	+++
Testes					++	+++++

Arbitrary scale: absence of labelling (−);weak (+) to high (+++++) concentrations; ne: not examined.

### Generation of MLF1-IP Gene KO Mice

**Figure 4:**
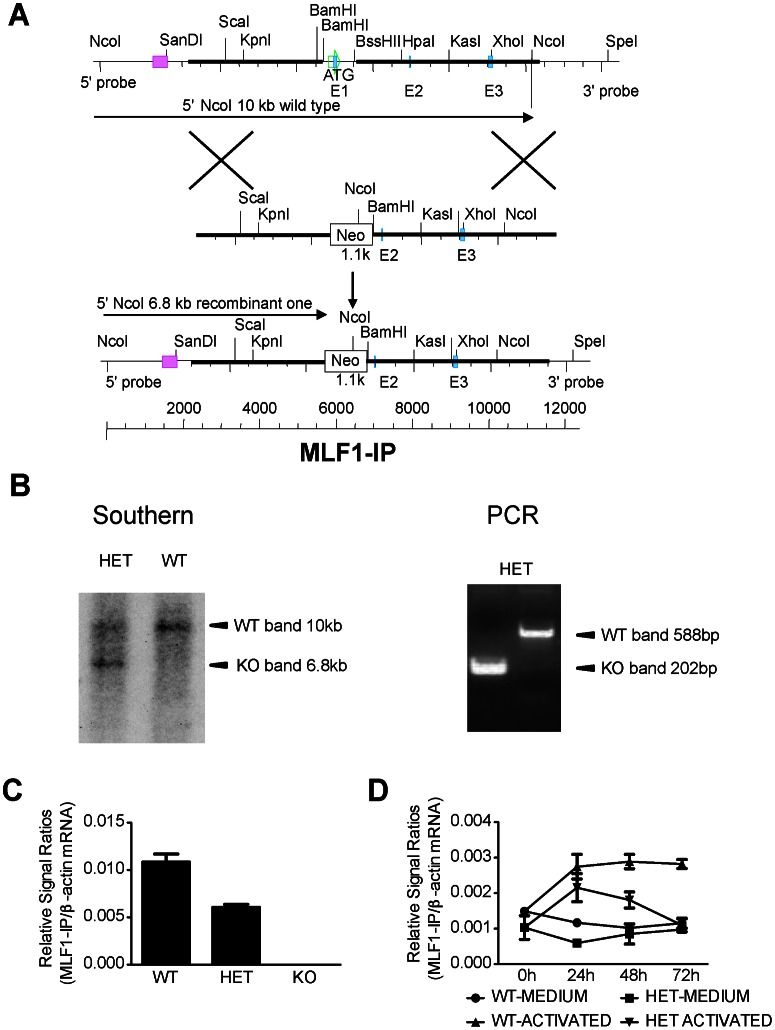
Generation of MLF1-IP KO mice. *A. Targeting strategy for generating MLF1-IP KO mice* The red square on the 5′ side of the mouse MLF1-IP WT genome represents the sequence used as a probe in Southern blotting for genotyping. *B. Genotyping of MLF1-IP mutant mice* Tail DNA was digested with NcoI, and analyzed by Southern blotting (left panel), with a 5′ probe whose location is indicated in A (red square). A 10-kb band representing the WT allele and a 6.8-kb band representing the recombinant allele are indicated by arrows. Ear lobe DNA sample was analyzed by PCR for routine genotyping (right panel). A 588-bp band representing the WT allele and a 202-bp band representing the recombinant allele are indicated by arrows. *C. MLF1-IP mRNA expression in MLF1-IP WT, HET and KO embryos* mRNA levels from e7.5 WT, HET and KO embryos were analyzed by RT-qPCR. The results are expressed as ratios of MLF1-IP versus β-actin signals with means±SD indicated. *D. MLF1-IP mRNA expression in WT and HET T cells upon activation.* T cells from WT and HET spleens were stimulated by solid-phase anti-CD3 mAb and anti-CD28 mAb (0.5 µg/ml and 4 µg/ml respectively for coating) for 0, 24, 48 or 72 h, and their MLF1-IP mRNA levels were quantified by RT-qPCR. The results are expressed as ratios of MLF1-IP versus β-actin signals with means±SD indicated.

We generated MLF1-IP KO mice to assess the role of MLF1-IP in live animals. The targeting strategy is depicted in Figure 4A. With the 5′ end probe, the WT allele after NcoI digestion presented a 10-kb band on Southern blotting, and the KO allele, a 6.8-kb band (Fig. 4A). Germline transmission was confirmed by Southern blotting of tail DNA, and WT and HET mice were thus identified (Fig. 4B, left panel). PCR was undertaken for routine genotyping of ear DNA. The WT allele presented a 588-bp band, and the KO allele, a 202-bp band (Fig. 4B, right panel).

To ascertain if MLF1-IP gene deletion affected its expression, we measured MLF1-IP mRNA levels of WT, HET and KO embryos (Fig. 4C). E7.5 HET embryos only expressed MLF1-IP mRNA at a 50% level of WT embryos, while KO embryos at that time totally lacked the mRNA.

MLF1-IP was previously selected for study because it was inducible after T-cell activation (data not reported). We activated T cells with solid-phase anti-CD3 and anti-CD28 monoclonal antibodies (mAbs), and quantified MLF1-IP mRNA at different time points (0, 24, 48 and 72 h after the initiation of culture). As shown in Fig. 4D, resting HET T cells (0 h, and cells cultured in medium for 24 h) expressed about 50% less MLF1-ip mRNA than their WT counterparts. After T cell activation, the MLF1-IP mRNA levels of both HET and WT T cells were upregulated with respect to their unstimulated controls, but the levels in HET T cells were about 50% that of the WT T cells.

The data in Figure 4C confirmed the gene deletion of MLF1-IP in KO embryos. Figure 4C and 4D also indicated that MLF1-IP expression was gene copy number-dependent, as HET cells expressed only one-half the level of MFL1-IP mRNA compared to WT cells.

### MLF1-IP KO is Lethal in Embryos

**Figure 5:**
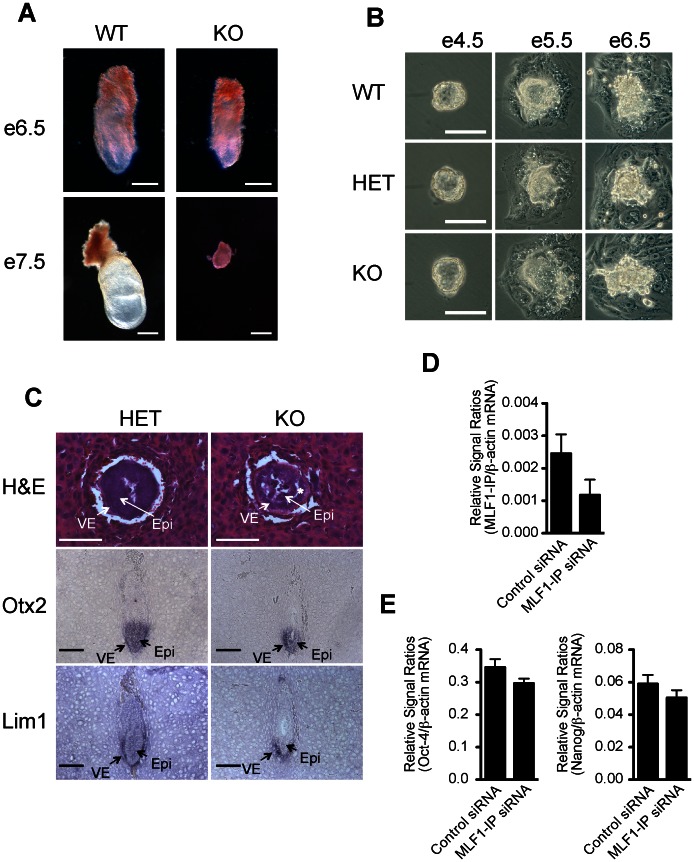
Development of WT, HET and KO embryos until e7.5. *A. Gross morphology of WT and KO mouse embryos.* E6.5 and e7.5 embryos dissected from decidua were photographed, and then genotyped by RT-qPCR. (Scale bar: 100 µm.). *B. Normal outgrowth of HET and KO embryos from e4.5 to e6.5 in vitro.* E3.5 embryos were harvested with M2 medium, cultured in M16 medium and photographed daily on e4.5, e5.5 and e6.5. Phase-contrast micrographs are shown. At the end of culture on e6.5, the embryos were genotyped by RT-qPCR. (Scale bar: 100 µm.). *C. Histology and differentiation marker expression at of foetuses e6.5.* Top row: Hematoxylin and eosin staining of e6.5 embryos. Middle row: Oxt2 in situ hybridization. Bottom row: Lim1 in situ hybridization. VE: visceral endoderm; Epi: epiblast; *: detachment of the epiblast from the surrounding visceral endoderm. (Scale bar: 100 µm.). *D. Knockdown of MLF1-IP mRNA expression in HET ES cells by siRNA according to RT-qPCR.* HET ES cells were transfected with MLF1-IP siRNA or control siRNA as indicated, and after 2 days, their MLF1-IP mRNA expression was assessed by RT-qPCR. *E. Oct-4 and Nanog mRNA expression in HET ES cells with MLF1-IP knockdown.* Oct-4 (left panel) and Nanog (right panel) expression levels of the cells from Fig. 5D were assessed by RT-qPCR.

We failed to generate any live MLF1-IP KO mice, nor could we obtain any KO embryos between e9 and birth. Systemic tracking of embryo genotype in different gestation stages revealed that intact KO embryos could only be found before and at e7.5 (Table 3) from MLF1-IP^+/−^ x MLF1-IP^+/−^ mating, and the frequency of KO embryo occurrence on e3.5, e6.5 and e7.5 was basically in agreement with Mendelian ratios. E3.5 KO embryos had no anomalies upon visual inspection (data not shown), while e6.5 KO embryos were moderately smaller than their WT counterparts (Fig. 5A). On e7.5, all KO embryos were much smaller size than their WT counterparts (Table 3 and Fig. 5A). We then cultured e3.5 embryos from HET male and female mating to observe their outgrowth *in vitro*. As depicted in Figure 5B, WT, HET and KO embryos manifested no discernible difference upon visual inspection 1 day after culture (e4.5), and their outgrowth seemed to be comparable until e6.5. All these data suggested major developmental blockage between e6.5 and e7.5 in the absence of MLF1-IP.

**Table 3:** Genotyping of embryos from MLF1-IP^+/−^ × MLF1-IP^+/−^ mating.

	Genotype			
Stage	+/+	+/−	−/−	Total
e3.5	3	18	9	30
e6.5	12	18	8^a^	41
e7.5	8	16	9^b^	33

a KO embryos were moderately smaller than WT ones.

b KO embryos drastically shrank to smaller sizes.

To better understand embryonic lethality around E6.5, we performed Hematoxylin and eosin staining which revealed a degeneration of the epiblast and its detachment from the surrounding visceral endoderm in e6.5 KO embryos (Fig. 5C, top row), while HET embryos, showed no such anomaly. *In situ* hybridization with an *Otx2* probe that marks both the epiblast and visceral endoderm revealed a hypocellular and disorganized epiblast tissue in KO but not HET embryos at e6.5 (Fig. 5C, middle row). *In situ* hybridization for *Lim1* that marks primarily the visceral endoderm revealed a somewhat thicker but otherwise normal visceral endoderm in e6.5 KO embryos, as compared to that in HET embryos (Fig. 5C, lower row).

These data indicate that MLF1-IP is vital in embryonic development, particularly for epiblast development around e6.5.

As shown in Figure 4C, MLF1-IP mRNA levels in heterozygous embryos was about a half of that in WT ones, but HET embryos develop normally. We failed to generate ES cells from KO embryos, but HET ES cells were generated. We wondered whether by further knocking down the MLF1-IP expression levels in the HET ES cells, we might reveal some anomaly in these cells in terms of their pluripotency. We transfected HET ES cells with MLF1-IP siRNA, and the mRNA knockdown was confirmed by RT-qPCR (Fig. 5D). The siRNA-or control siRNA-transfected ES cells were cultured for 2 days, and their expression of Oct-4 and Nanog, two pluripotent markers was assessed by RT-qPCR [17]. As shown in Figure 5E, no apparent differences in Oct-4 and Nanog expression between the MLF1-IP and control siRNA-transfected HET ES cells were observed. Thus, probably the remaining MLF1-IP is sufficient to maintain the pluripotency of ES cells.

### No Detectable Anomalies in Cell Sub-populations in Lymphoid Organs and Bone Marrow of MLF1-IP HET Mice

As MLF1-IP was prominently expressed in the thymus between e15.5 and p10, and was up-regulated in adult T cells upon their activation, we set out to investigate whether reduced MLF1-IP expression causes immune system abnormalities. HET mice were used for this purpose because no KO mice could be produced. HET mice were viable and fertile with not visible abnormalities on either gross or anatomical visual inspection. No lymphoid organ anomalies were apparent in HET mice in terms of size, weight and cellularity (data not reported).

**Figure 6:**
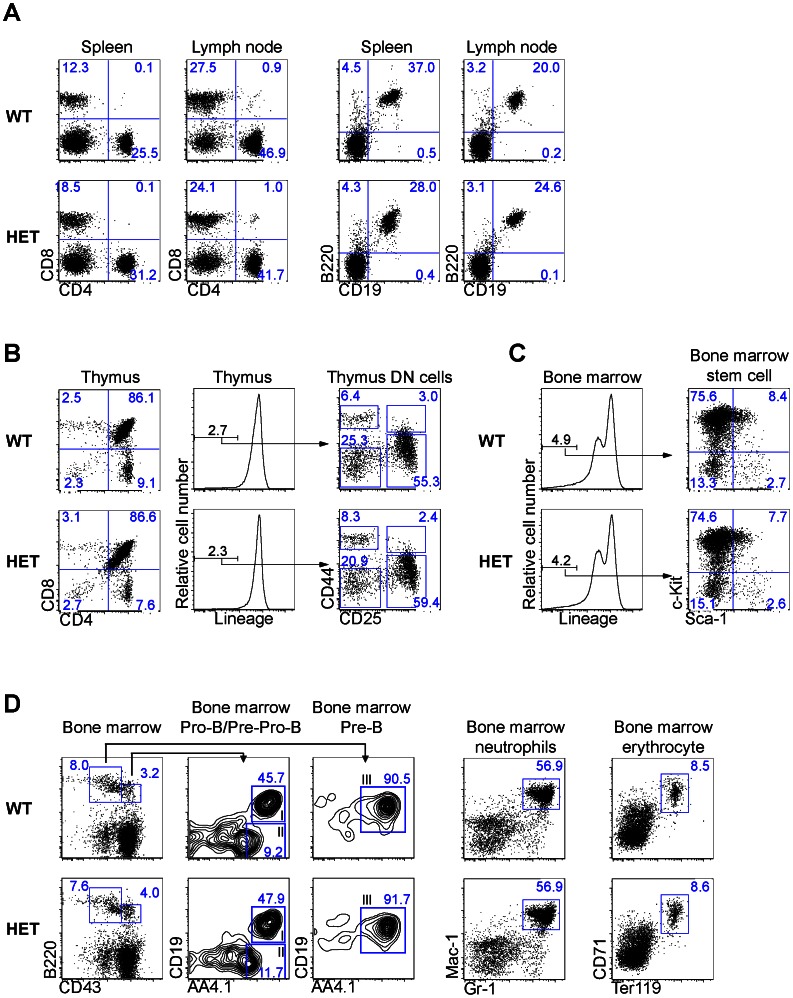
Sub-populations of lymphocytes in lymphoid organs of MLF1-IP HET mice. Cells of hematopoietic origin in lymphoid organs and bone marrow were analyzed by flow cytometry. The percentages of key sub-populations are indicated. The experiments were repeated at least 3 times, and representative histograms provided. *A. T- and B -cell sub-populations in the spleen and lymph nodes.* CD4 and CD8 T-cell sub-populations in WT and HET spleens, lymph nodes and thymus were analyzed by 2-color flow cytometry. The B-cell population in the spleen and lymph nodes was analyzed according to B220 and CD19 expression by 2-color flow cytometry. Percentages are indicated. *B. Thymocyte sub-populations.* Thymocytes from WT and HET mice were stained with CD4 or CD8 for 2-color flow cytometry, or with lineage markers (CD3ε, CD8β, TCRβ, CD11b, CD45R, B220, Ly6C, Ly6G, Ter-119), CD25 and CD44 for 3-color flow cytometry. Lineage^-^ cells were gated and analyzed for their CD25 and CD44 expression. *C. Lineage-negative bone marrow cell sub-populations.* WT and HET bone marrow cells were stained with lineage markers, c-Kit and Sca-1for 3-color flow cytometry. Lineage- cells were gated, and analyzed for their c-Kit^+^Sca-1^+^ stem cell population. *D. Pre-B, pro-B, pre-pro-B cell sub-populations and myeloid cell in bone marrow.* WT and HET bone marrow cells were stained with B220, CD43, CD19 and AA4.1 and analyzed by 4-color flow cytometry for B220^+^CD43^+^CD19^-^AA4.1^+^ pre-pro-B cell (region II in column 2) B220^+^CD43^+^CD19^+^AA4.1^+^ pro-B cell (region I in column 2) and B220^+^CD43^-^CD19^+^AA4.1^+^ pre-B cell (region III in column 3) populations. Bone marrow cells were also stained with Mac-1 and Gr-1 (column 4) for analysis of mature Mac-1^+^Gr-1^+^ neutrophils, or stained with Ter-119 and CD71 for analysis of CD71^+^Ter119^+^ erythroid precursor cells.

T, B, CD4 and CD8 sub-populations in the spleen and lymph nodes of HET mice were comparable to those in WT controls (Fig. 6A), as were thymocyte sub-populations, such as CD4CD8 double-negative, CD4CD8 double-positive, CD4 single-positive and CD8 single-positive cells (Fig. 6B).

We also assessed the Lin^-^c-Kit^+^Sca-1^+^ stem cell population in bone marrow, but no significant difference between HET and WT mice was evident (Fig. 6C). Furthermore, populations of B220^+^CD43^+^CD19^+^AA4.1^+^ pro-B cells, B220^+^CD43^-^CD19^+^AA4.1^+^ pre-B cells, Mac-1^+^Gr-1^+^ bone marrow myeloid cells, and CD71^+^Ter119^+^ erythroid precursors were comparable in the bone marrow of HET and WT mice (Fig. 6D).

### Assessment of HET and WT T-cell Functions

**Figure 7:**
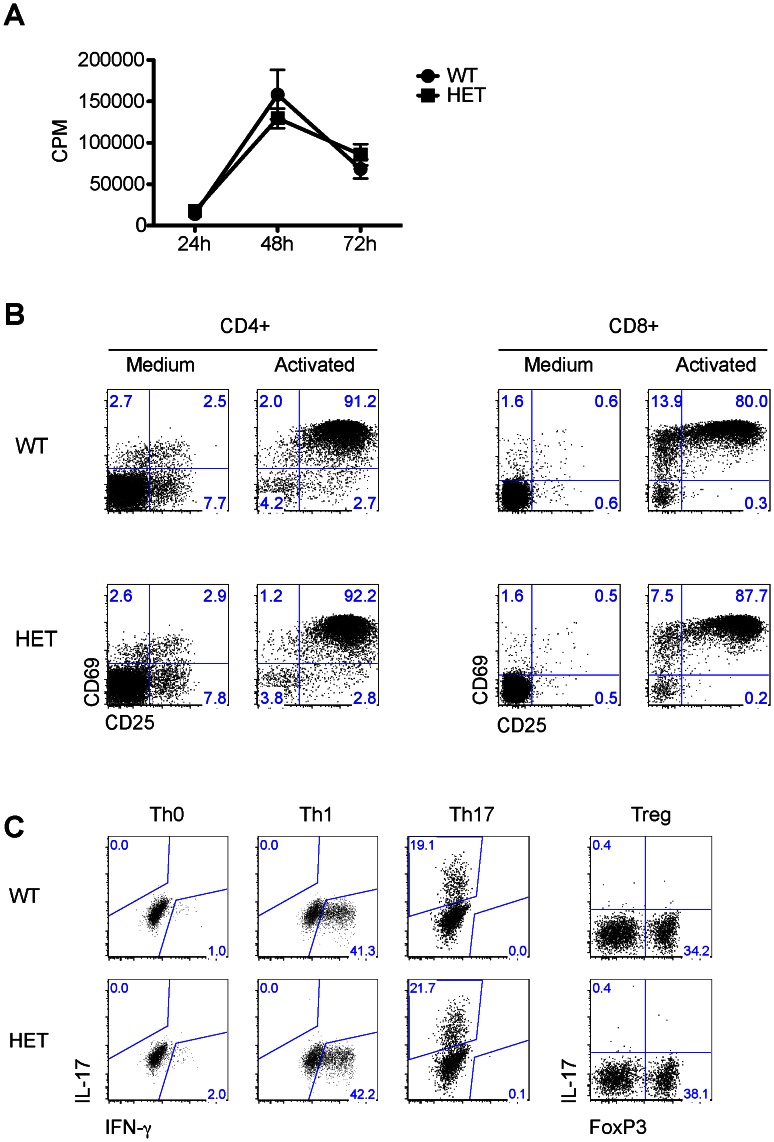
MLF1-IP^+/−^ T-cell functions *in vitro*. All experiments in this figure were repeated at least twice and representative data are presented. *A. T-cell proliferation.* WT and HET spleen T cells were stimulated with solid-phase anti-CD3 mAb plus anti-CD28 mAb (0.5 µg/ml and 4 µg/ml respectively for coating) and pulsed with ^3^H-thymidine for 16 h before harvesting. ^3^H-thymidine uptake was measured at 24, 48 and 72 h after the initiation of culture. Samples were tested in triplicate, and cpm means±SD were reported. *B. C69 and CD25 expression on activated WT and HET T cells.* WT and HET T cells were stimulated overnight by solid-phase anti-CD3 mAb plus anti-CD28 mAb (0.5 µg/ml and 4 µg/ml respectively for coating). CD69 and CD25 expression on CD4 (left panel) and CD8 (right panel) T cells was measured by 2-color flow cytometry. *C. Naïve CD4 cell differentiation in vitro.* WT and HET Naïve CD4 cells were cultured under conditions favouring Th1 (second column), Th17 (third column), or Treg (last column) cell differentiation for 3 days. Cells without differentiation cytokines (TH0, first column) were included for comparison. Their intracellular cytokine expression was determined by flow cytometry. Experiments were repeated twice, and representative data reported.

Although HET T cells only expressed MLF1-IP mRNA at about 50% the WT level upon activation by anti-CD3 and CD28 mAbs on solid phase, they proliferated as well as WT T cells (Fig. 7A). Activation markers, such as CD25 and CD69, were up-regulated in HET CD4 and CD8 T cells comparably to their WT counterparts (Fig. 7B). Furthermore, HET-naïve CD4 T cells could differentiate into Th1, Th17 and Treg cells as efficiently as WT T cells (Fig. 7C). Therefore, it appears that 50% of normal MLF1-IP expression is sufficient to maintain T-cell proliferation and function.

## Discussion

MLF1-IP came to our attention about 10 years ago when we screened genes whose expression was up-regulated during T-cell activation, but little was known about its functions at that time. Several years later, its functions in mitosis were reported by several groups (3–6). Our ISH analysis of its temporal and spatial expression during ontogeny in mice by and large concurs with its reported function in mitosis in that regions in organs containing fast-proliferating cells at any given ontogeny stage expressed high MLF1-IP mRNA levels. However, such was not always the case. For example, on p10, moderately high MLF1-IP mRNA expression was still obvious in the retina and cerebellum, although these regions do not have fast growing cells at that stage. This raises an intriguing question: does MLF1-IP have additional functions besides those in mitosis.

Our study, for the first time, examines whether MLF1-IP is irreplaceable or its function can be compensated by other molecules. With complete MLF1-IP deletion, this question was clearly answered in the KO mouse model. At least until close to e6.5, MLF1-IP KO embryos were comparable to WT controls in regard to size and morphology. Multiple rounds of proliferation were carried out from inception to the time point near e6.5, starting from a fertilized egg to an embryo of about 0.5 mm in length containing several hundred thousand cells. Obviously, proliferation could go on without MLF1-IP, and was not absolutely needed for mitosis. On the other hand, it is obvious that without MLF1-IP, embryonic development cannot go beyond e6.5, indicating that certain vital developmental events around this time absolutely require MLF1-IP. We attempted to generate KO ES cells from e3.5 blastocysts. From 100 blastocysts, 50–60 ES cell lines were obtained, but all of them were either WT or HET. Taken together, these findings suggest that MLF1-IP is essential in optimizing the embryonic developmental program: cells might develop without MLF1-IP, but once the condition is stringent, such as during ES cell derivation *in vitro* or near e6.5 *in vivo*, cells without MLF1-IP will fail to develop further. It seems different types of cells in the early development require different levels of MLF1-IP expression; the epiblast is more sensitive to the lack of MLF1-IP at e6.5. We conclude that MLF1-IP is crucially required during embryonic development. However, the fact that a fertilized egg can grow into several hundred thousand cells without MLF1-IP raises questions as to whether such irreplaceability is simply due to its roles in the proliferation program, or due to its vital roles in other particular cellular processes such as epiblast development around e6.5.

Another useful piece of information from our study is that 50% of normal MLF1-IP expression is sufficient during ontogeny and immunity. HET mice were alive and fertile, with no visible anomalies. Their lymphoid organs and cells all developed normally, and no defective T cell function was observed *in vitro* based on our assays. Thus, normal MLF1-IP expression level has ample buffering latitude for its functions.

In summary, our study, for the first time, demonstrated the irreplaceability of MLF1-IP during mouse embryonic development. It also raised an intriguing possibility that the molecule might have functions unrelated to proliferation, based on its expression pattern in the CNS and successful progression of embryonic development until e6.5.
